# Selective vagus–recurrent laryngeal nerve anastomosis guided by intraoperative neuromonitoring: evidence of lateral motor fiber clustering in the vagus nerve

**DOI:** 10.3389/fendo.2026.1845996

**Published:** 2026-06-17

**Authors:** Jiedong Kou, Daqi Zhang, Le Zhou, Shijie Li, Tie Wang, Peiyao Wang, Zihan Zhao, Gianlorenzo Dionigi, Carla Colombo, Yishen Zhao, Hui Sun

**Affiliations:** 1 Division of Thyroid Surgery, The China-Japan Union Hospital of Jilin University, Jilin Provincial Key Laboratory of Thyroid Disease, Jilin Provincial Precision Medicine Laboratory of Molecular Biology and Translational Medicine on Differentiated Thyroid Carcinoma, Changchun, Jilin, China; 2 Department of Pathophysiology and Transplantation, University of Milan, Milan, Italy

**Keywords:** intraoperative neuromonitoring, nerve regeneration and functional recovery, recurrent laryngeal nerve injury, selective vagus–recurrent laryngeal nerve anastomosis, thyroid surgery

## Abstract

**Objective:**

Direct anastomosis (DA) is the standard approach after recurrent laryngeal nerve (RLN) transection but is often not feasible due to excessive tension. This experimental study evaluated a novel intraoperative neuromonitoring (IONM)-guided selective vagus-recurrent laryngeal nerve anastomosis (SVRA) technique and compared its immediate electrophysiologic performance with DA in a porcine thyroid surgery model.

**Methods:**

18 transected nerves from 9 pigs were randomized to DA or SVRA (9 nerves per group). In the SVRA group, low-current IONM was used to map vagus nerve (VN) motor fibers innervating laryngeal musculature; these fibers were selectively dissected and anastomosed to the transected RLN. In the DA group, end-to-end RLN neurorrhaphy was performed under microscopy. Electromyography (EMG) amplitudes and latencies were recorded at baseline and serially up to 2 hours after anastomosis; hemodynamic parameters were monitored to assess the safety of VN manipulation.

**Results:**

VN motor fibers innervating the laryngeal muscles were predominantly localized to the lateral VN and were mostly concentrated in a single strand. After anastomosis, both techniques yielded early EMG recovery, with post-anastomotic amplitudes often exceeding 50% of baseline. A cross-innervation model (left VN to right RLN) produced immediate EMG responses approaching baseline and bilateral vocal fold activation. Moreover, when the anastomosed nerve was pulled, the EMG amplitude varied with the alteration of the relative position of the fiber components at the two severed ends. VN dissection did not cause clinically relevant changes in blood pressure or oxygen saturation, and only minor, non-significant heart rate increases were observed.

**Conclusion:**

IONM-guided SVRA enables selective recruitment of VN motor fibers for targeted RLN reconstruction while largely preserving VN trunk integrity. These findings support SVRA as a physiologically grounded and technically feasible reconstructive option that can achieve acute electrophysiological recovery when tension precludes DA during thyroid surgery–related RLN transection, although long-term functional reinnervation remains to be established.

## Highlights

- Low-current IONM maps a consistent posterolateral cluster of laryngeal motor fibers in the cervical vagus nerve.
- Selective vagus–RLN anastomosis using this cluster achieves immediate EMG recovery comparable to direct anastomosis.
- The technique preserves most vagal trunk fibers and may be a function-preserving alternative when tension precludes direct RLN repair.

## Introduction

Transection of the recurrent laryngeal nerve (RLN) may result in ipsilateral vocal fold paralysis or impaired phonatory motility. Delayed treatment often leads to a poor prognosis and even lifelong disability (1–5). Several reconstructive strategies have been proposed to restore RLN continuity and function, including direct anastomosis (DA), free nerve graft anastomosis (FNGA), ansa cervicalis–RLN anastomosis (ARA), and vagus nerve (VN)–RLN anastomosis (VRA). Experimental and clinical studies have shown that these techniques can promote nerve regeneration and improve phonatory outcomes (6–10). However, each method has important limitations: DA is often precluded by excessive tension; FNGA requires donor nerve harvest; ARA may provide suboptimal matching of motor fibers; and classic VRA sacrifices the entire VN trunk, raising concerns about systemic autonomic effects.

Anatomical and electrophysiological data indicate that only a fraction (approximately 20%-25%) of VN fibers are efferent motor fibers that innervate the laryngeal musculature (11). Clinical intraoperative neuromonitoring (IONM) studies have further suggested that specific VN segments yield higher electromyography (EMG) amplitudes and can be selectively targeted for RLN reinnervation, with EMG recovery documented after reconstruction (12). These observations provide a biological rationale for a more selective, function-preserving approach to VN–RLN anastomosis.

Porcine models, which closely replicate human laryngeal and cervical nerve anatomy, offer a robust platform to test advanced nerve repair strategies and to correlate detailed neuroanatomy with intraoperative electrophysiological findings. Building on this background, the present study systematically maps VN motor fiber distribution using low-current IONM and evaluates a novel selective vagus-recurrent laryngeal nerve anastomosis (SVRA) technique in comparison with DA in a porcine model. The aim is to define electrophysiologically guided, clinically instructive protocols for tension-free, selective RLN reinnervation that preserve VN function and may be translated into thyroid surgery practice.

## Materials and methods

### Ethical approval

All experiments were conducted in accordance with the ethical policies and procedures of the Ethics Committee of the China-Japan Union Hospital of Jilin University (approval number NO.2022-KYYS-078). All authors of this study strictly adhered to the ARRIVE Guidelines (13).

### Animal model and surgical preparation

The experimental model used domestic “Parma” pigs aged 6.5-8.0 months and weighing 28.9-30.2 kg. Animals were anesthetized and monitored according to institutional standards. The RLN transection model was created by sharply dividing the RLN with surgical scissors under magnification. Vital signs, including heart rate, femoral arterial blood pressure (continuous invasive monitoring), and oxygen saturation, were recorded throughout the procedure.

### IONM stimulation device

A standard IONM system (Medtronic) was used. A unipolar ball probe delivered electrical stimulation to the VN and RLN, and EMG signals were recorded via an endotracheal tube with integrated surface electrodes positioned at the level of the vocal folds. To minimize artifacts, no intraoperative rotation or compression of the endotracheal tube was allowed once optimal contact was confirmed. Low-current neurostimulation was used to map VN motor fibers innervating the laryngeal musculature. Low current was defined as the smallest current at which the EMG amplitude (>100 μV) could be detected within a specific quadrant of the nerve cross-section. Nerve fibers capable of eliciting reproducible laryngeal EMG responses under minimal-current stimulation were considered candidate motor fascicles contributing to laryngeal innervation.

### Study design and randomization

**Figure 1:**
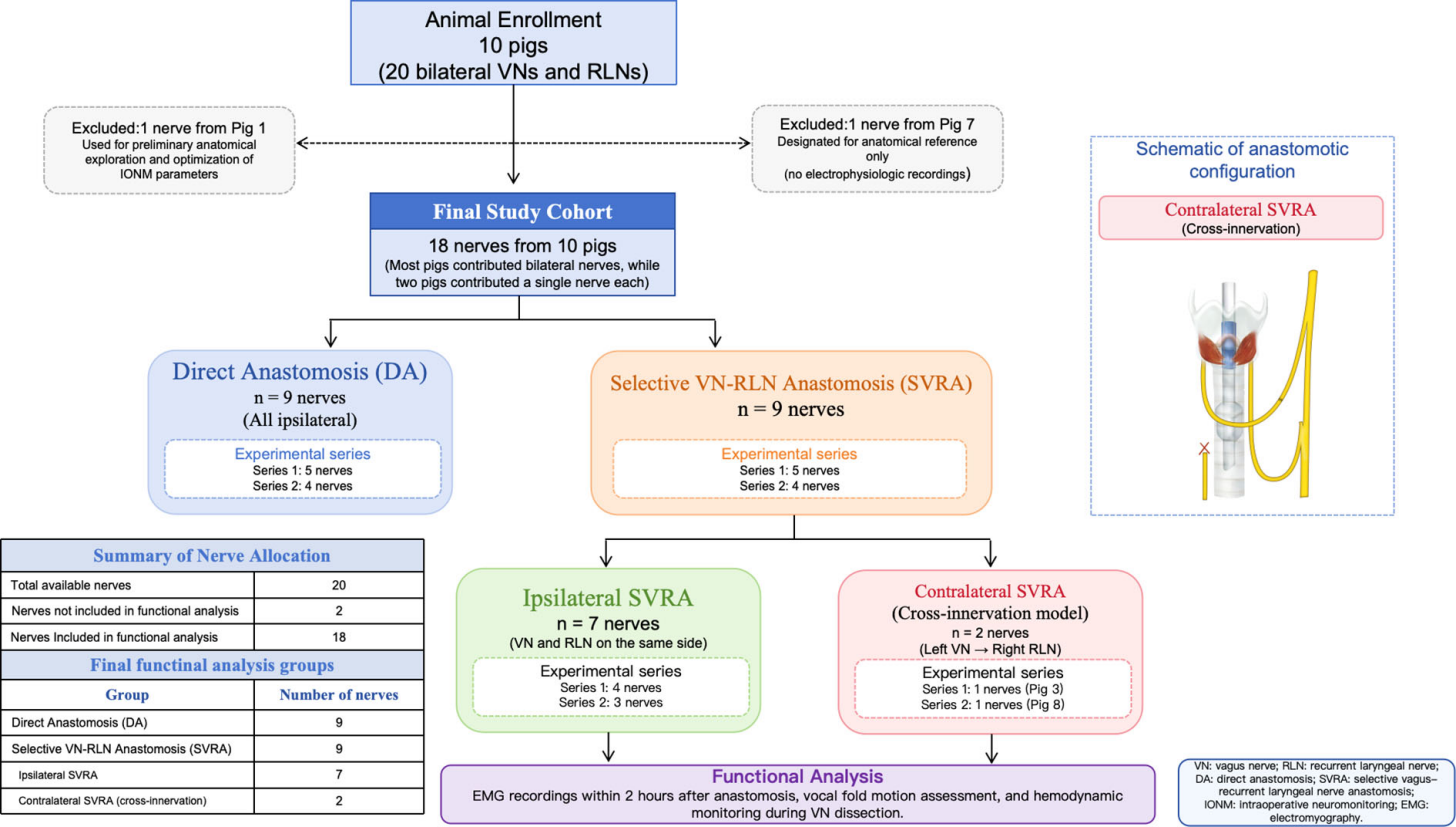
Schematic flow diagram of study design, nerve allocation, and anastomotic configurations. A total of 10 pigs (20 bilateral VNs and RLNs) were included in the study. One nerve from pig 1 was used for preliminary anatomical exploration and optimization of IONM parameters, and one nerve from pig 7 was designated for anatomical reference only; both were excluded from functional electrophysiological analysis. The remaining 18 nerves from 10 pigs were included in the final analysis, with most animals contributing bilateral nerves and two animals contributing a single nerve each. These nerves were allocated to either direct anastomosis (DA, n = 9; all ipsilateral RLN repairs) or selective vagus–recurrent laryngeal nerve anastomosis (SVRA, n = 9). Within the SVRA group, seven reconstructions were performed ipsilaterally, and two were configured as contralateral cross-innervation models (left VN to right RLN). Experiments were conducted in two sequential series. Functional assessment included intraoperative EMG recordings within 2 hours after anastomosis, qualitative laryngeal movement evaluation using intraoperative laryngoscopy when feasible, and hemodynamic monitoring during VN dissection. RLN, recurrent laryngeal nerve; VN, vagus nerve; EMG, electromyography; IONM, intraoperative neuromonitoring; DA, direct anastomosis; SVRA, selective vagus–recurrent laryngeal nerve anastomosis.

A total of 20 bilateral VNs and RLNs from 10 pigs were initially available for study. One nerve from pig 1 was used for preliminary anatomical exploration and optimization of IONM parameters prior to formal experimentation. In addition, one nerve from pig 7 was designated for anatomical reference only and was not included in electrophysiological outcome analysis. The remaining 18 nerves from pigs constituted the formal study cohort and were allocated to either the SVRA group or the DA group, with nine nerves in each group. Among the nine nerves assigned to the SVRA group, seven underwent ipsilateral selective VN-RLN anastomosis, while two were intentionally reconstructed using a contralateral cross-innervation model (left VN to right RLN) to further explore electrophysiological feasibility. Experiments were conducted in two sequential phases, and all eligible nerves were ultimately included in the final functional analysis (**Figure 1**).

### Electrophysiologic mapping of vagus nerve motor fibers

**Figure 2:**
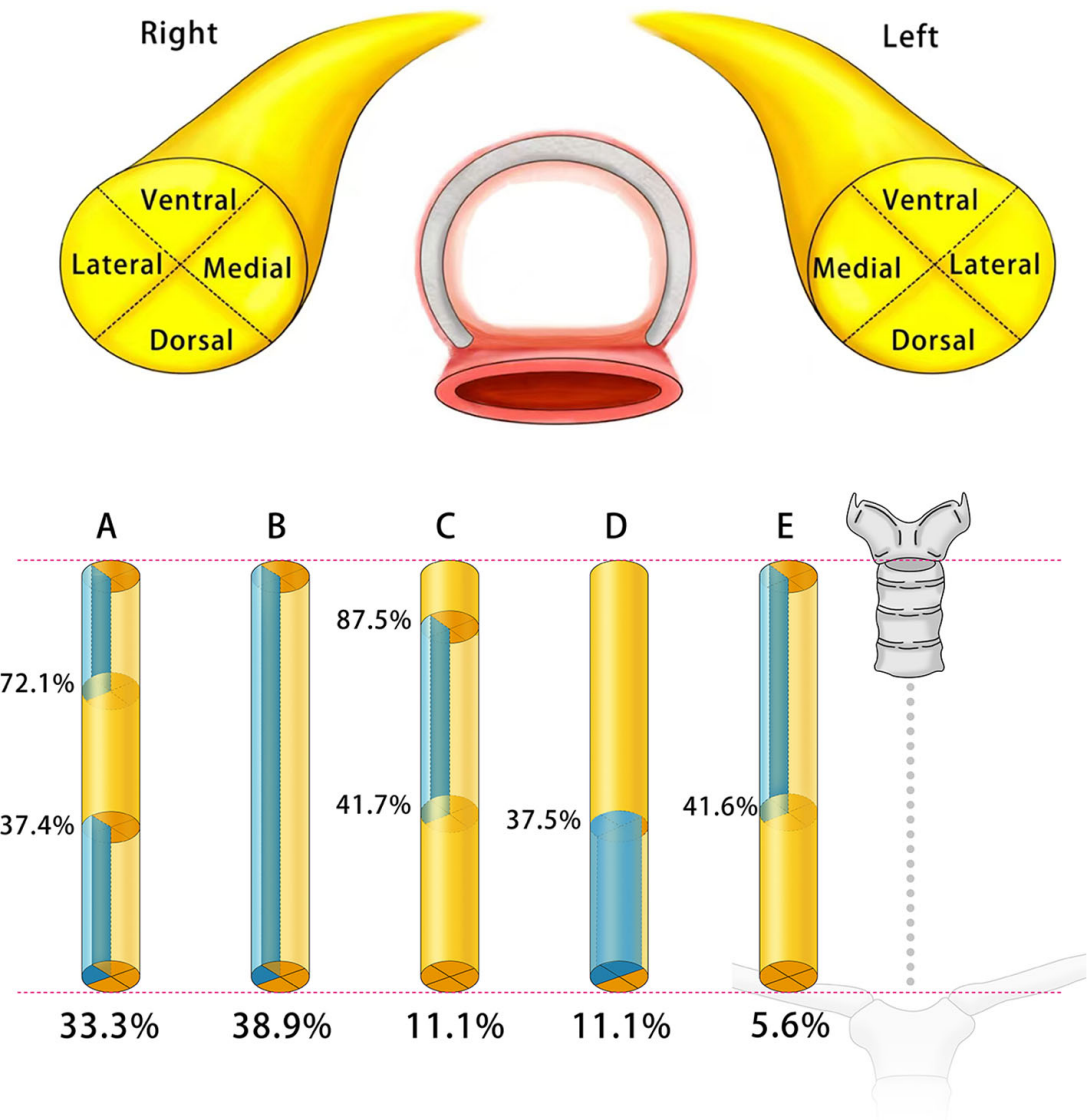
Low-current mapping of vagus nerve motor fibers and classification of distribution patterns. EMG responses were recorded at four standardized stimulation sites (ventral, dorsal, medial, lateral) around the VN cross-section to localize RLN-related motor components. The side adjacent to the trachea is defined as medial, and the opposite side as lateral (upper panels). Based on serial low-current stimulation from the upper sternal margin to the RLN laryngeal entry point, longitudinal VN motor fiber distribution was categorized into five types (lower panels). Yellow denotes a dispersed distribution, where EMG signals are elicited from multiple quadrants, while blue indicates a concentrated distribution, with motor fibers clustered in a restricted region of the nerve. VN, vagus nerve; RLN, recurrent laryngeal nerve.

**Figure 3:**
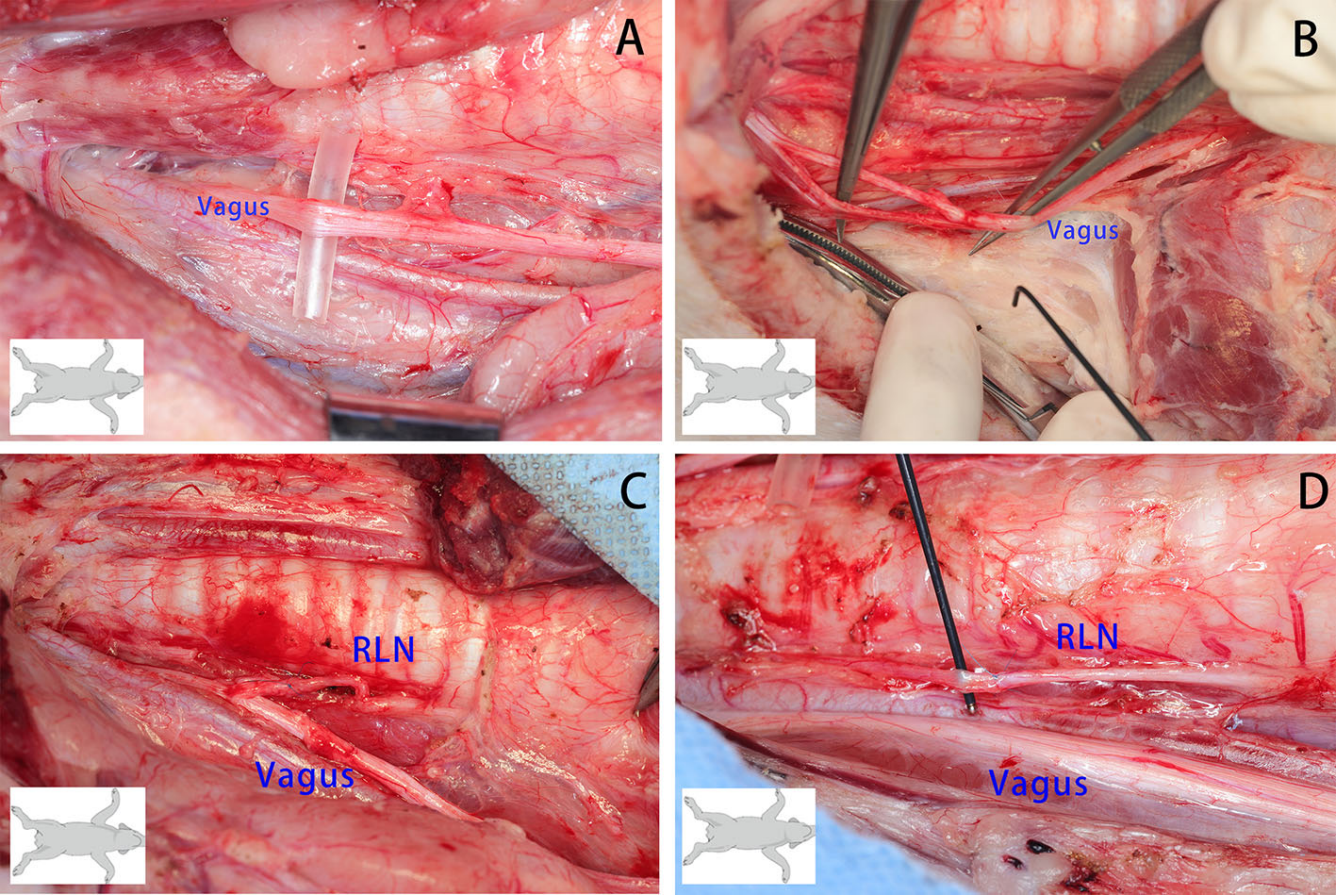
Neuroanatomical separation and anastomosis of the vagus and recurrent laryngeal nerves. To prevent current spread to the RLN during VN stimulation, the VN was circumferentially dissected, and an insulating rubber tube was placed beneath the nerve to electrically isolate the stimulation site from surrounding tissues **(A)**.Using low-current IONM “navigation,” VN motor fiber components were identified and microsurgically separated from the main trunk, resulting in two bundles; EMG activity was preserved with amplitudes comparable to baseline in the motor bundle, while the companion bundle became EMG-silent **(B)**. For SVRA, the EMG-active VN branch was transected at a point that allowed tension-free coaptation and sutured end-to-end to the ipsilateral transected RLN **(C)**. For DA, the RLN was sharply divided, and the two stumps were directly reapproximated with end-to-end neurorrhaphy **(D)**. VN, vagus nerve; RLN, recurrent laryngeal nerve; EMG, electromyography.

To localize RLN-related components within the VN, EMG responses were systematically recorded at four stimulation sites on the VN cross-section- ventral, dorsal, medial (tracheal side), and lateral (away from the trachea)- starting from the upper sternal border and proceeding cranially toward the RLN entry into the larynx (**Figure 2**). After 360°exposure of the VN, an insulating rubber tube was placed beneath the nerve to prevent current spread to the RLN (**Figure 3A**). At each level, stimulation began at 0.01 mA and was increased stepwise until an EMG signal was detected at one or more quadrants; the stimulation site, current intensity, EMG amplitude, and latency were recorded. The probe was then advanced in 0.5-cm increments toward the head, and the same mapping protocol was repeated up to the level of the RLN laryngeal entry point or until further VN exposure was not feasible. Functionally identified fascicles associated with reproducible laryngeal EMG activation were selectively dissected for SVRA reconstruction while preserving the majority of the remaining VN trunk.

### Selective vagus–recurrent laryngeal nerve anastomosis technique

After VN mapping, the site with the minimal stimulation current still capable of eliciting a robust EMG response was identified as the presumed motor fiber-rich segment. Under low-current IONM guidance, this segment of VN fibers was microsurgically dissected from the VN trunk, separating it into two bundles (one EMG-active, one EMG-silent) as confirmed by repeat stimulation (**Figure 3B**). The EMG-active VN branch was then transected at a point that allowed tension-free coaptation with the ipsilateral transected RLN. End-to-end neurorrhaphy between the VN motor branch and the RLN was performed using 8–0 microsutures (monofilament, absorbable) under a 2.5× surgical microscope (ZTGX, XTS-4C), which was similar to the method used in previous experiments (4), ensuring precise alignment of the perineurium and avoiding rotation or torsion at the anastomotic site (**Figure 3C**). EMG recordings of the reconstructed pathway were obtained immediately, and then at 30 minutes, 1 hour, 1.5 hours, and 2 hours after anastomosis.

### Direct recurrent laryngeal nerve anastomosis technique

In the DA group, the functionally intact RLN was sharply divided, and the two stumps were approximated in an end-to-end fashion using 8–0 microsutures (monofilament, absorbable) under the same microscope, restoring anatomical continuity without interposition grafts. Particular care was taken to align the nerve ends without excessive tension and to avoid compression or kinking at the repair site (**Figure 3D**). EMG amplitudes and latencies were recorded using the same time schedule as in the SVRA group.

### Cross-innervation model

**Figure 4:**
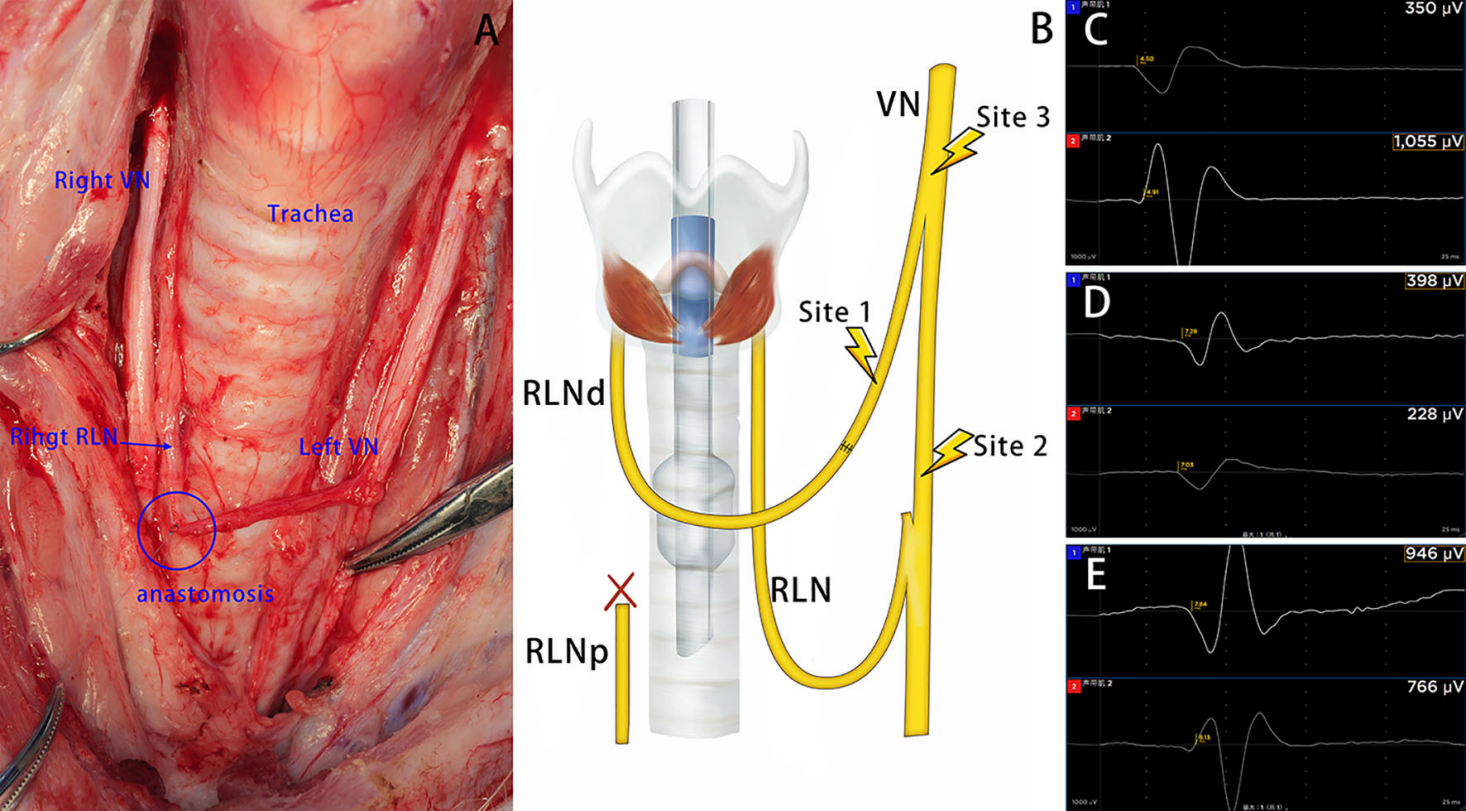
Cross-innervation of the left vagus nerve to the right recurrent laryngeal nerve and EMG responses at different stimulation sites. After dissection of the left VN in pigs no. 3 and 8, VN motor fibers innervating the laryngeal muscles were selectively isolated and anastomosed to the right transected RLN, while the remaining VN trunk remained connected to the left RLN **(A)**. Following anastomosis, electrical stimulation near the VN dissociation point evoked both ipsilateral and contralateral EMG signals, with distinct response patterns depending on the stimulation site along the reconstructed pathway **(B)**. Stimulation at site 1 activated only the right side, inducing a right EMG amplitude of 1055 μV **(C)**. Stimulation at site 2, applied to the distal portion of the dissociated VN segment within the separation zone, elicited a left EMG amplitude of 398 μV **(D)**. When a 3.0 mA stimulus was delivered at site 3, at the proximal end of the dissociation region, bilateral EMG responses were recorded with amplitudes of 946 μV and 766 μV on the left and right sides, respectively **(E)**. A, intraoperative image; B, schematic diagram. RLN, recurrent laryngeal nerve; VN, vagus nerve; EMG, electromyography.

To further characterize the electrophysiologic feasibility and functional potential of selective VN motor fiber transfer, two contralateral cross-innervation models were intentionally established within the SVRA group (pigs 3 and 8). In these cases, motor fibers selectively identified from the left VN were dissected and anastomosed to the transected right RLN, while preserving the native ipsilateral left VN-RLN pathway. This configuration established a new left VN–right RLN pathway in parallel with the original left VN–left RLN system. EMG responses were recorded, and laryngeal movement was qualitatively assessed using intraoperative laryngoscopy during stimulation of different sites along the dissociated VN segment and proximal trunk (**Figure 4**; **Supplementary Video 1**).

### Post-anastomotic functional assessment

For each anastomosed nerve, baseline EMG amplitude and latency were recorded before transection. After SVRA or DA, EMG responses were obtained at predefined time points using the same stimulation parameters and electrode configuration. Changes in EMG amplitude relative to baseline quantified immediate functional recovery. In selected cases, the reconstructed nerve was gently manipulated or pulled to evaluate the impact of relative fiber position and tension on EMG amplitude and signal stability. When feasible, intraoperative laryngoscopy was performed during nerve stimulation to qualitatively assess arytenoid movement and subtle vocal fold motion; this assessment was observational and not intended as a formal functional endpoint.

### Hemodynamic monitoring and safety evaluation

In three pigs, detailed hemodynamic monitoring was performed during VN dissection and anastomosis to assess the cardiovascular safety of VN manipulation. Continuous measurements of blood pressure, heart rate, and oxygen saturation were recorded and analyzed at baseline and during key procedural steps.

### Sample size and power analysis

This study was designed as an exploratory experimental investigation to characterize the feasibility and immediate electrophysiologic effects of SVRA compared with DA in a porcine model. A formal *a priori* power calculation was not feasible because robust preliminary data on expected differences in EMG amplitude between SVRA and DA were not available at the time of study planning. Based on logistical constraints and ethical considerations regarding large-animal experiments, we selected a convenience sample of 10 pigs, yielding 18 analyzable nerves (9 per group) after exclusion of one nerve used for anatomical optimization and one reserved for anatomical reference. This sample size was considered sufficient to: (1) allow repeated within-nerve EMG measurements over time; (2) detect large effect sizes in EMG recovery (e.g., post-anastomotic amplitudes ≥50% of baseline) between techniques; and (3) provide preliminary estimates of variability to inform future, adequately powered confirmatory studies. The results of this work should therefore be interpreted as hypothesis-generating rather than definitive.

### Statistical analysis

Quantitative data are expressed as mean ± standard deviation or median (interquartile range), as appropriate. Categorical variables are presented as frequency and percentage. The quantitative data were analyzed by t test, Mann-Whitney U test or Kruskal-Wallis H test. *P* values were two-tailed and *P* < 0.05 was considered statistically significant. Statistical analysis was performed using IBM SPSS 23.0.

## Results

All nerves showed baseline EMG amplitudes greater than 500 μV, indicating adequate monitoring sensitivity according to international standards (14–16). The average length of the exposed nerves was 6.9 ± 2.1 cm. The location of the stimulation site is expressed as a percentage of the total length (from the upper edge of the sternum to the level of the laryngeal entry point of the RLN) of a distance (from the recording point to the upper margin of the sternum).

### Longitudinal (axial) distribution patterns of VN motor fibers

The response current required to elicit EMG signals ranged from 0.02 to 0.4 mA. Using the minimal effective current, EMG responses were obtained with lateral, ventral, medial, and dorsal stimulation in 17, 11, 10, and 5 nerves, respectively. Based on these patterns, VN motor fiber distribution along the longitudinal axis was classified into five types (**Figure 2**):

Class A (n=6): From 0% (upper sternal edge) to 37.4% ± 23.32% (mean ± SD) of the VN length, motor fibers were concentrated on the lateral side; from 37.4% ± 23.32% to 72.1% ± 18.76% of the distance, they were scattered; from 72.1% ± 18.76% to 100% (laryngeal entry point) distance, they again concentrated laterally.

Class B (n=7): Neuromotor components remained continuously concentrated on the lateral side from the upper sternal edge to the RLN entry level; only one animal showed additional medial segment involvement.

Class C (n=2): Motor fibers were circumferentially distributed near the upper sternal margin (0-41.7% ± 11.8%), concentrated laterally in the mid-segment (41.7% ± 11.8%-87.5% ± 5.9%), and again circumferential near the RLN entry level (87.5% ± 5.9%-100%).

Class D (n=2): Motor fibers were concentrated on the lateral or superior aspect from 0-37.5% ± 41.3%, then became scattered toward the RLN entry level (37.5% ± 41.3%–100%).

Class E (n=1): Motor fibers were initially scattered from 0-41.6%, then gradually condensed into a single cord up to the RLN entry level (41.6%-100%).

Overall, 77.8% (14/18) of nerves showed convergence of motor fibers into a single cord near the RLN entry point. These findings indicate that VN motor fibers display reproducible longitudinal clustering, particularly near the laryngeal entry zone, which is favorable for selective anastomosis planning.

### Cross-sectional localization of VN motor fibers

Motor fibers innervating the laryngeal musculature were predominantly located in the lateral quadrant of the VN, although additional scattered fibers were present in other quadrants. Under minimal-current stimulation, EMG signals were detected in the lateral portion of the VN in 94.4% (17/18) of nerves, while ventral and dorsal responses were observed in 11 and 5 nerves, respectively (**Table 1**). These data confirm that the lateral VN is the principal reservoir of laryngeal motor fibers and represents the most promising target area for selective VN–RLN anastomosis. Taken together, these data indicate that laryngeal motor fibers are predominantly located in the lateral (posterolateral) quadrant of the VN in more than 90% of nerves, which has direct implications for where to dissect and coapt the fascicle during SVRA.

**Table 1:** Electromyographic responses at various vagus nerve stimulation sites during low-current mapping.

Category	Ventral with signal	Dorsal with signal	Medial with signal	Lateral with signal	Total
Number of nerves *	11(61.1%)	5(27.8%)	10(55.6%)	17(94.4%)	18(100%)
Left nerve	6	2	4	9	9
Right nerve	5	3	6	8	9

*, the proportion of the nerves with detectable EMG signal at this part of the cross-section in the total number of nerves.

### Immediate electrophysiologic effects of anastomosis

A total of 18 nerves underwent reconstruction using either DA (n = 9) or SVRA (n = 9) across two experimental series. Within the SVRA group, seven nerves underwent ipsilateral VN–RLN reconstruction, while two were intentionally configured as contralateral cross-innervation models (left VN to right RLN).

In the first experimental series (10 nerves) (**Table 2**), early EMG recovery within 2 hours was observed in 1/5 nerves in the DA group, with an amplitude of 350 μV (56.1% of baseline) at 1.5 hours post-anastomosis. In the SVRA group, 1/4 ipsilateral reconstructions demonstrated EMG recovery within 2 hours, with an amplitude of 450 μV (85.7% of baseline) at 30 minutes, while the remaining nerves showed no detectable EMG signal within this time window.

**Table 2:** Early EMG signal recovery after nerve reconstruction using DA and SVRA.

Parameter	DA group (n = 9)	SVRA group (n = 9)
Nerves with detectable EMG signal	5 (55.6%)	6 (66.7%)
Ipsilateral reconstruction	9	7
Contralateral reconstruction	—	2
EMG-positive nerves (ipsilateral)	5	4
EMG-positive nerves (contralateral)	—	2

Contralateral reconstruction refers to selective VN motor fibers from the left vagus nerve anastomosed to the right RLN (cross-innervation model).

In the second series (8 nerves) (**Table 2**), after optimization of fascicular alignment and minimization of tension, all reconstructed nerves (4 DA and 4 SVRA) demonstrated detectable EMG signals near the repair site, with amplitudes exceeding 50% of baseline.

### Contralateral cross-innervation findings

Two contralateral SVRA reconstructions were established to further explore cross-side electrophysiological connectivity (**Figure 4A**). In these models, selectively identified motor fibers from the left VN were anastomosed to the transected right RLN while preserving the native ipsilateral VN–RLN pathway. Immediate EMG responses were recorded following reconstruction. Stimulation at different sites along the dissected VN segment elicited variable activation patterns, including unilateral and bilateral EMG responses. Stimulation at site 1 (**Figure 4B**) induced a right EMG amplitude of 1055 μV (93.6% of baseline) with a latency of 4.91 ms (**Figure 4C**); stimulation at site 2 produced a left EMG amplitude of 398 μV (33.1% of baseline) with a latency of 7.28 ms (**Figure 4D**). Stimulation at site 3 elicited bilateral EMG responses with amplitudes of 946 μV and 766 μV on the left and right sides, respectively (**Figure 4E**). These findings demonstrate that selective VN motor fibers can support cross-innervation in specific configurations. In these cases, stimulation of the reconstructed pathway was associated with visible arytenoid movement and subtle vocal fold motion.

### Technical factors affecting signal recovery

Across both DA and SVRA reconstructions, EMG amplitude was sensitive to subtle changes in the relative position, angular alignment, and tension at the anastomotic site. Minor adjustments in fascicular alignment could significantly enhance signal amplitude, whereas excessive traction occasionally reduced or transiently abolished EMG responses. These observations highlight the critical importance of precise microsurgical technique and optimal tension control for achieving reliable electrophysiological conduction following nerve reconstruction.

### Hemodynamic safety of VN dissection

Blood pressure, heart rate, and oxygen saturation were monitored during VN manipulation in three experimental pigs. The intraoperative changes in vital signs in the experimental pigs are shown in **Table 3**; **Supplementary Table 1**. Blood pressure and blood oxygen saturation remained stable throughout the experimental procedure. Two pigs had slightly increased heart rate during VN dissociation, but the difference was not statistically significant with *t* test (*P* > 0.05). Oxygen saturation remained stable in all experimental pigs throughout the procedure and was between 99 and 100. Overall, VN dissociation and selective fiber harvesting did not induce clinically relevant cardiovascular instability, supporting the short-term safety of the SVRA approach in this model.

**Table 3:** Changes of vital signs of experimental pigs during operation.

Time	Systolic pressure (mm Hg)	Diastolic pressure (mm Hg)	HR (times/minute)	SaO2
At the beginning of anesthesia	71	45	83	100
10 minutes after anesthesia	99	63	70	100
20 minutes after anesthesia	84	53	70	100
30 minutes after anesthesia	83	49	68	100
5 minutes before dissociation of VN	80	50	95	99
At dissociation of VN	75	48	87	99
5 minutes after dissociation of VN	76	52	81	99
10 minutes after dissociation of VN	85	54	89	99
15 minutes after dissociation of VN	90	59	101	99
1 hour after dissociation of VN	82	56	113	99
Before VN dissociation, mean ± SD	83.4 ± 10.1	52 ± 6.8	77.2 ± 11.6	100(99.5,100)
After VN dissociation, mean ± SD	81.6 ± 6.3	53.8 ± 4.1	94.2 ± 12.8	99(99,99)
*P*-value(Before vs after dissociation)	0.744	0.626	0.059	NA

At the beginning of anesthesia, 10, 20 and 30 minutes after anesthesia, and 5 minutes before dissociation of VN all belongs to Before VN dissociation; 5,10,15minutes and 1 hours after dissociation of VN belongs to After VN dissociation.

## Discussion

### Rationale for selective VN–RLN anastomosis

DA is not feasible in all cases of RLN injury, especially when tumor invasion or tissue loss creates excessive tension at the repair site (12). In such situations, surgeons must use alternative reinnervation strategies such as VRA, FNGA, or ARA, each with specific advantages and drawbacks (8, 17, 18). The main effects of four anastomosis methods and the number of implementation cases conducted by different researchers during 1998–2020 is summarized in **Table 4** (6, 8–10, 12, 17–25). The VN, as the proximal parent nerve of the RLN, inherently contains the motor fibers that drive laryngeal musculature, making it an anatomically and functionally attractive donor for RLN reconstruction (26, 27). Selective VN–RLN anastomosis, guided by IONM, aims to exploit this relationship by recruiting only those VN fibers that innervate the vocal folds, while preserving most VN trunk function. Our porcine model, which closely reproduces human laryngeal and cervical nerve anatomy, provides a suitable platform to investigate this concept in a controlled experimental setting.

**Table 4:** Research reports on the implementation of different anastomotic methods.

Anastomotic methods	Number of studies	Number of cases	Years	Subjects	Neuroanastomosis effect
DA	11	124	1998–2020	Patients	VHI/GRBAS/MPT can be improved by immediate repair after nerve transection, and multiple studies have shown that postoperative recovery is independent of anastomosis mode
ARA	9	508	1998–2020		
FNGA	6	25	1998–2020		
VRA	2	14	1998–2016		

DA, direct anastomosis; FNGA, free nerve graft anastomosis; ARA, ansa cervicalis–RLN anastomosis; VRA, vagus nerve (VN)–RLN anastomosis.

### Segmental and cross-sectional distribution of VN motor fibers

In this study, low-current IONM mapping demonstrated that VN motor fibers innervating the vocal folds are not randomly distributed but show reproducible longitudinal and cross-sectional clustering, with predominant localization in the lateral VN quadrant and convergence into a single cord near the RLN entry point in 77.8% of nerves. This distribution pattern challenges the traditional assumption that mixed nerve fibers invariably emit branches on the side closest to their target and instead supports a more complex, “twisted-braid” architecture of motor fascicles within the VN. From a surgical perspective, these findings underscore that blind or empirical VN division is suboptimal; instead, targeted low-current mapping is essential to identify the fascicles most likely to contribute to laryngeal motor function and to maximize the success of SVRA.

### “Twisted-braid” organization and implications for NRLN

The classification of longitudinal VN patterns into five types revealed that, particularly in Class A nerves, motor fibers may be dispersed in mid-segments and progressively re-cluster laterally toward the laryngeal entry zone. This “twisted-braid” phenomenon led us to hypothesize that VN motor components possess an intrinsic ability to reorganize at multiple cervical levels, potentially contributing to the embryologic formation of non-recurrent laryngeal nerves (NRLNs) when RLN components do not follow the typical course around the subclavian artery. In this context, SVRA can be interpreted as the deliberate creation of an “artificial NRLN,” re-routing VN motor fibers toward the RLN stump in a manner that respects the natural growth and rearrangement patterns of mixed motor fibers. Such a physiologic rationale may partly explain the robust electrophysiologic recovery observed in selected SVRA configurations in our experiments.

### Feasibility of non-ipsilateral VN–RLN reinnervation

The cross-innervation model, in which left VN motor fibers were anastomosed to the right RLN while the left VN trunk remained connected to the left RLN, provides additional insights into the flexibility of this system. Stimulation of the dissociated VN branch supplying the right RLN (R system) produced EMG amplitudes on the right side comparable to baseline RLN values, whereas stimulation of the remaining trunk (L system) yielded lower amplitudes, likely reflecting partial loss of left-sided motor fibers. These findings suggest that a subset of RLN motor fibers remains within the VN trunk even after selective harvesting and can continue to drive ipsilateral effector muscles, while the transferred fascicle functions predominantly as a “current source” for the reconstructed contralateral RLN. Although this non-ipsilateral configuration is of theoretical interest and demonstrates the capacity for bilateral vocal cord activation from a single VN, it is unlikely to be clinically preferable to an ipsilateral SVRA due to the higher risk of functional imbalance and the greater complexity of the reconstruction.

### Mechanistic considerations of current transmission and misdirected regeneration

Our observations that EMG amplitudes across the anastomosis varied with subtle changes in nerve alignment and tension, and could be transiently abolished with altered coaptation positioning, highlight the critical importance of precise fascicular coaptation for preserving conduction. We hypothesize that early current transmission across the repair site is mediated not only by structural continuity but also by dynamic transmembrane ion gradients in Schwann cells at the anastomosis, with synchronized ion fluxes between proximal and distal segments maintaining functional conduction before full axonal regeneration occurs. These electrophysiologic phenomena must be interpreted in light of the well−described problem of misdirected regeneration in laryngeal nerves, where mixed reinnervation of adductor and abductor muscles may limit restoration of normal vocal fold mobility despite substantial improvement in phonatory quality (4, 28–30).In clinical translation, SVRA may therefore be most valuable for improving voice and airway protection rather than guaranteeing complete recovery of physiologic vocal fold motion.

### Reverse tracer strategy and advantages over conventional VRA

The “reverse tracer” concept applied in this study – using low−current IONM to identify RLN−related motor fibers within the VN at clinically accessible levels – provides a practical roadmap for selective anastomosis in the operative field between the upper sternum and the laryngeal entry point. Our experimental and clinical observations, including a case of VN sheath tumor in which only one specific VN branch elicited laryngeal EMG responses, support the notion that laryngeal motor fascicles are regionally concentrated and can be selectively targeted. Unlike conventional VRA, in which the VN is transected in its entirety with potential systemic autonomic consequences, SVRA preserves the majority of VN trunk fibers while redirecting only a functionally characterized motor component to the RLN stump. This function−preserving design may reduce the risk of cardiovascular or visceral side effects and offers a more refined, anatomically guided alternative to classic VRA in selected patients.

### Safety of VN dissociation and hemodynamic impact

Hemodynamic monitoring in a subset of animals showed that VN dissection and selective fiber harvesting were associated with stable blood pressure and oxygen saturation, and only minor, non−significant increases in heart rate during manipulation. These data suggest that, at least in the short term and within the extent of dissection performed in this model, SVRA can be carried out without clinically relevant cardiovascular instability. Because the VN contains important autonomic fibers involved in cardiovascular and visceral regulation, preservation of the majority of the VN trunk during selective fascicular dissection may help reduce the risk of major autonomic dysfunction. Nonetheless, given the wide autonomic distribution of the VN, careful intraoperative monitoring and conservative dissection remain essential in any future clinical application.

### Strengths and limitations

This study has several strengths. First, it integrates detailed anatomical mapping with real−time IONM to characterize VN motor fiber distribution and guide selective anastomosis, providing mechanistic insight and a reproducible technical framework. Second, the use of a porcine model with laryngeal anatomy similar to humans allows for realistic simulation of thyroid surgery–related RLN injuries and reconstructions, including both ipsilateral and cross−innervation scenarios. Third, the study systematically compares DA and SVRA using quantitative EMG endpoints and standardized time points, offering initial evidence that both techniques can restore acute electrophysiologic conduction across the anastomosis and achieve early EMG signal recovery when tension and alignment are optimized, although these findings do not yet confirm true regenerative reinnervation. In addition, the use of low-current IONM–guided selective fascicular mapping allowed targeted recruitment of candidate laryngeal motor fibers while preserving most of the remaining VN trunk, thereby potentially reducing the extent of vagal injury compared with conventional whole-VN transfer approaches.

Several limitations must also be acknowledged. The sample size is relatively small, reflecting the ethical and logistical constraints of large−animal research, and limits the statistical power to detect subtle differences between DA and SVRA or to generalize the observed patterns to all anatomical variants. The follow−up period was restricted to acute, intraoperative EMG measurements; long-term functional outcomes such as sustained vocal fold motion, regenerative reinnervation patterns, synkinetic reinnervation, and histologic remodeling were not assessed, limiting interpretation of whether early EMG recovery reflects durable functional restoration or only transient electrophysiological conduction. As an animal study, the anatomical and neurophysiological characteristics of porcine nerves do not fully replicate human variability, and extrapolation to clinical practice should therefore be cautious.

### Clinical implications and future directions

Despite these limitations, our findings support IONM-guided SVRA as a physiologically grounded and technically feasible proof-of-concept strategy for RLN reconstruction in situations where direct tension-free repair is not possible. The concept of SVRA originated from the clinical challenge of reconstructing RLN defects when direct tension-free repair is not feasible, particularly in cases requiring extensive tumor resection. Compared with conventional whole-VN transfer techniques, selective low-current IONM-guided fascicular transfer may offer a more conservative strategy by preserving the majority of the VN trunk while selectively recruiting candidate laryngeal motor fibers. They also provide a detailed electrophysiologic map that may guide selective fascicular targeting in future clinical applications. Importantly, immediate postoperative EMG recovery should not be interpreted as definitive evidence of axonal regeneration or long-term functional reinnervation, but rather as confirmation of restored neural continuity and short-term electrophysiological conduction across the reconstructed pathway. Accordingly, the clinical implications of these findings should be interpreted with caution, given that the present results are derived from an experimental large-animal model with short-term observations. In addition, the observed laryngeal movements during intraoperative laryngoscopy were qualitative and limited by surgical exposure, and therefore do not constitute a formal assessment of functional vocal fold recovery. Although no clinically significant short-term hemodynamic instability was observed in this study, the possibility of delayed or subtle autonomic dysfunction related to VN manipulation cannot be completely excluded. Future research should focus on larger animal cohorts with long-term follow-up, including laryngoscopic, histologic, and functional assessments, to determine whether early electrophysiological continuity ultimately translates into durable functional reinnervation. Prospective clinical studies will be needed to determine whether SVRA, applied under strict IONM guidance and careful vagus nerve preservation, can improve voice and swallowing outcomes in patients requiring RLN sacrifice for oncologic clearance.

## Conclusion

In this porcine model, both DA and SVRA produced immediate or early recovery of EMG signals across the repair, indicating rapid restoration of functional conduction. Low-current IONM reliably identified VN motor fibers innervating the laryngeal musculature, which were predominantly clustered in the lateral portion of the VN and could be selectively dissected for targeted RLN reinnervation. The reverse-tracer strategy allowed these mapped VN motor components to be anastomosed to the transected RLN, promoting re-establishment of both anatomical continuity and functional connectivity. VN dissociation and selective fiber harvesting were not associated with significant hemodynamic instability in this model, suggesting short-term procedural safety. Taken together, these findings support DA and IONM-guided SVRA as biologically plausible reconstructive options when RLN sacrifice is required, particularly in situations where tension precludes conventional DA during thyroid cancer surgery.

## Data Availability

The original contributions presented in the study are included in the article/**Supplementary Material**. Further inquiries can be directed to the corresponding authors.
