# Supplementary material for: Selective vagus–recurrent laryngeal nerve anastomosis guided by intraoperative neuromonitoring: evidence of lateral motor fiber clustering in the vagus nerve
Source: Front Endocrinol (Lausanne). 2026 Jun 17;17:1845996. doi: 10.3389/fendo.2026.1845996 (PMC13318641; doi:10.3389/fendo.2026.1845996)
Supplement: Supplementary file 1 [file Table1.docx]

Supplementary Table 1: Heart rate before and after vagus nerve dissociation

|  | Experiment pig no.1 | Experiment pig no. 2 | Experiment pig no.3 |
| --- | --- | --- | --- |
| Before VN dissociation, mean±SD | 77.2±11.6 | 89.3±4.3 | 83.8±18.3 |
| After VN dissociation, mean±SD | 94.2±12.8 | 86.3±4.1 | 103.8±3.3 |
| *P* value(Before vs after dissociation) | 0.059 | 0.209 | 0.075 |
